# Unilateral pigmented paravenous retinochoroidal atrophy with acute angle-closure glaucoma: a case report

**DOI:** 10.1186/s12886-023-02922-4

**Published:** 2023-05-08

**Authors:** Kun Lv, Zhiqiao Liang, Kangyi Yang, Xuanzhu Chen, Yao Ma, Huijuan Wu

**Affiliations:** 1 Department of Ophthalmology, Peking University People’s Hospital, Beijing, China; 2 Eye Diseases and Optometry Institute, Beijing Key Laboratory of Diagnosis and Therapy of Retinal and Choroid Diseases, College of Optometry, Peking University Health Science Center, Beijing, China

**Keywords:** Pigmented paravenous retinochoroidal atrophy, Acute angle-closure glaucoma, Unilateral

## Abstract

**Background:**

Pigmented paravenous retinochoroidal atrophy (PPRCA) is an uncommon fundus disease characterized by perivenous aggregations of pigment clumps and retinochoroidal atrophy distributed along the retinal veins. We report a Chinese female case of unilateral PPRCA with acute angle-closure glaucoma (AACG).

**Case presentation:**

A 50-year-old Chinese female presented with vision loss and elevated intraocular pressure (IOP) in the right eye and then underwent trabeculectomy. She referred to our clinic for further evaluation and treatment. The funduscopic examination revealed grayish retinochoroidal atrophy and osteocyte-like pigment clumping lesions along the retinal veins and peripapillary preretinal hemorrhage in the right eye. The patient also presented with AACG in the same eye on the basis of past medical history of acute attack, shallow anterior chamber depth (ACD), narrow angle showed by ultrasound biomicroscopy (UBM) and glaucomatous neuropathy identified by optical coherence tomography (OCT). Other examinations like fluorescein fundus angiography (FFA), electroretinogram (ERG) and electrooculography (EOG) all confirmed the aforementioned diagnose.

**Conclusion:**

PPRCA is a rare disease, uncommon in females and symmetrical in both eyes. We present a rare case of unilateral PPRCA accompanied with AACG.

## Background

Pigmented paravenous retinochoroidal atrophy (PPRCA) is a rare fundus disease in which there is aggregations of osteocyte-like pigmentation associated with perivenous retina and choroid atrophy [1]. It usually occurs in a bilaterally symmetric form and are often diagnosed during a routine examination in asymptomatic patients based on typical fundus appearance [2].

In this case report, we presented a unique case of unilateral PPRCA in a middle-aged Chinese female who was diagnosed with PPRCA by accident when acute angle-closure glaucoma (AACG) attacked.

## Case report

A 50-year-old female complained of loss of vision in her right eye accompanied by ocular pain, headache, nausea and vomiting. The intraocular pressure (IOP) was 55 mmHg in the right eye and 11 mmHg in the left eye at the time of her visit to the local clinic. The fundus could not be seen clearly due to corneal edema. She denied any remarkable systemic, ocular disease history and was not taking any medication. She later underwent trabeculectomy in the right eye there and the IOP decreased to normal level and the cornea became clear. However, the visual acuity remained poor and she was referred to our glaucoma clinic for further evaluation and treatment.

At the time of current visit, visual acuity was 20/500 in the right eye and 20/20 in the left eye, with IOPs of 16.0 mmHg and 11.3 mmHg respectively. Slit lamp revealed the cornea was clear and the anterior chamber was free of cells or flare in right eye and the pupil diameter was 4 mm with extensive posterior synechia and no response to the light. A diffuse superior subconjunctival filtering bleb and a peripheral iridotomy at 9 o’clock were observed.

**Fig. 1:**
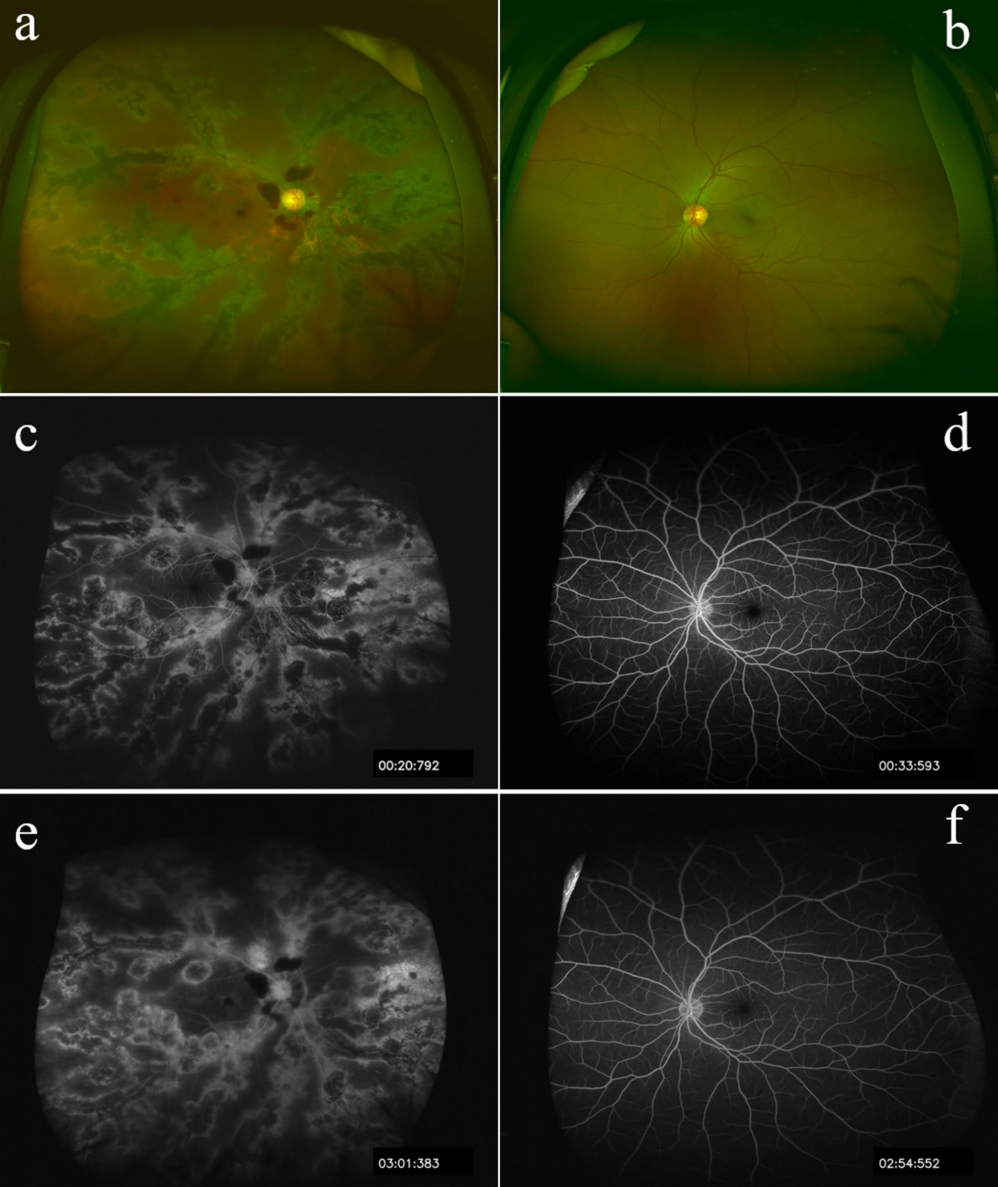
(a) Ultrawide-field fundus photography showed grayish retinochoroidal atrophy and osteocyte-like pigment clumping lesions along the retinal veins and peripapillary preretinal hemorrhage right eye. (b) Normal fundus in the left eye. (c) Early fluorescein fundus angiography (FFA) showed transmitted hyperfluorescence consistent with retinal pigment epithelium (RPE) degeneration in the right eye with more extensive areas of choriocapillaris atrophy. (d) Early FFA result was within normal limits in the left eye. (e) Late FFA of the right eye showed weak fluorescence and delay in venous filling in the atrophic areas. (f) Late FFA result was within normal limits in the left eye

Ultrawide-field fundus photography showed grayish retinochoroidal atrophy and osteocyte-like pigment clumping lesions along the retinal veins and peripapillary preretinal hemorrhage in the right eye (Fig. 1a), without any signs of inflammatory responses of the retina and vitreous body. The fellow eye examination was within normal limits (Fig. 1b).

**Fig. 2:**
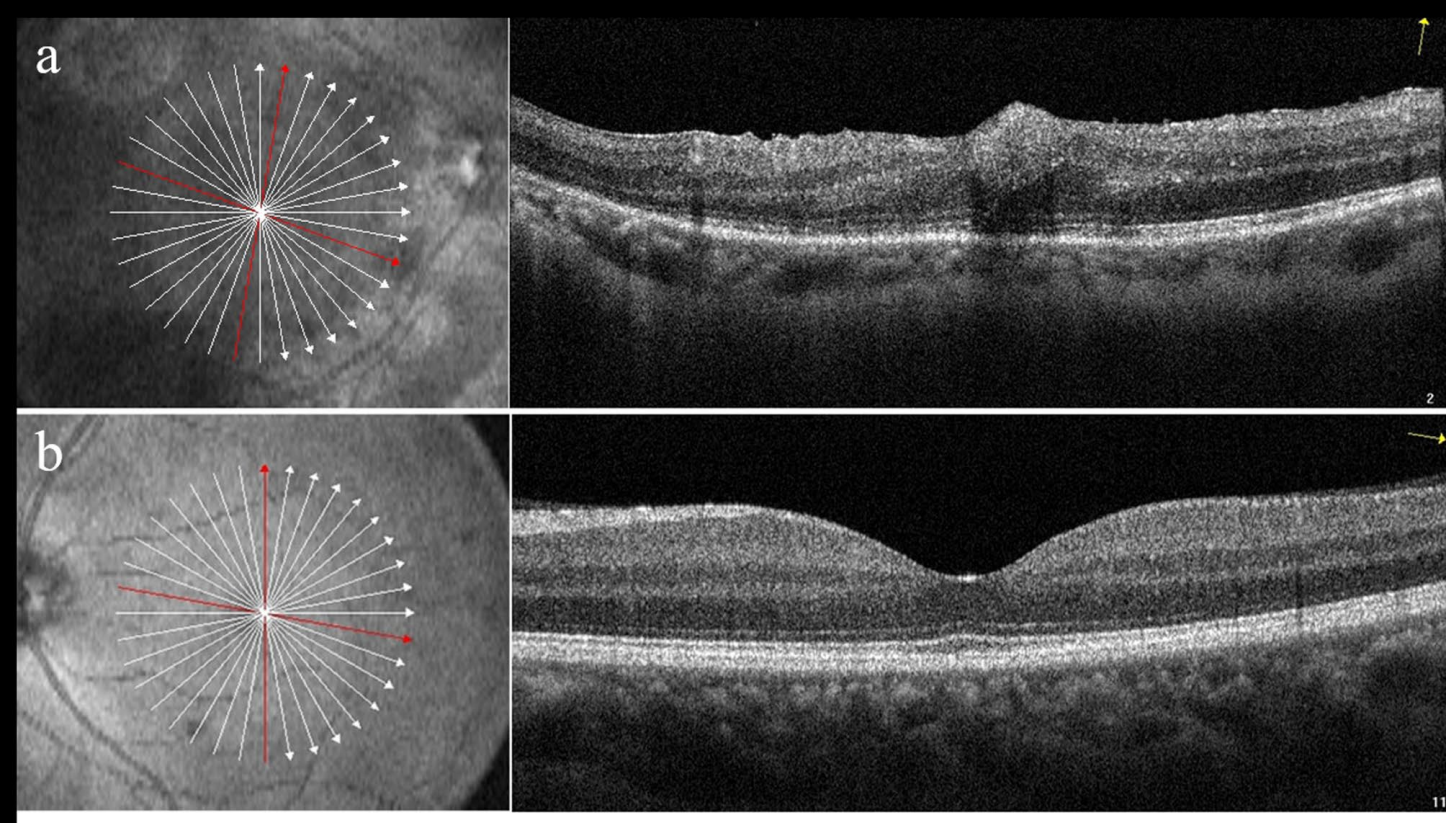
(a) Macular optical coherence tomography (OCT) findings of the right eye revealed a hyperreflective superficial lesion at the fovea consistent with a preretinal hemorrhage. Atrophy of the retinal pigment epithelium (RPE) is shown. (b) Normal OCT result of the left eye

**Fig. 3:**
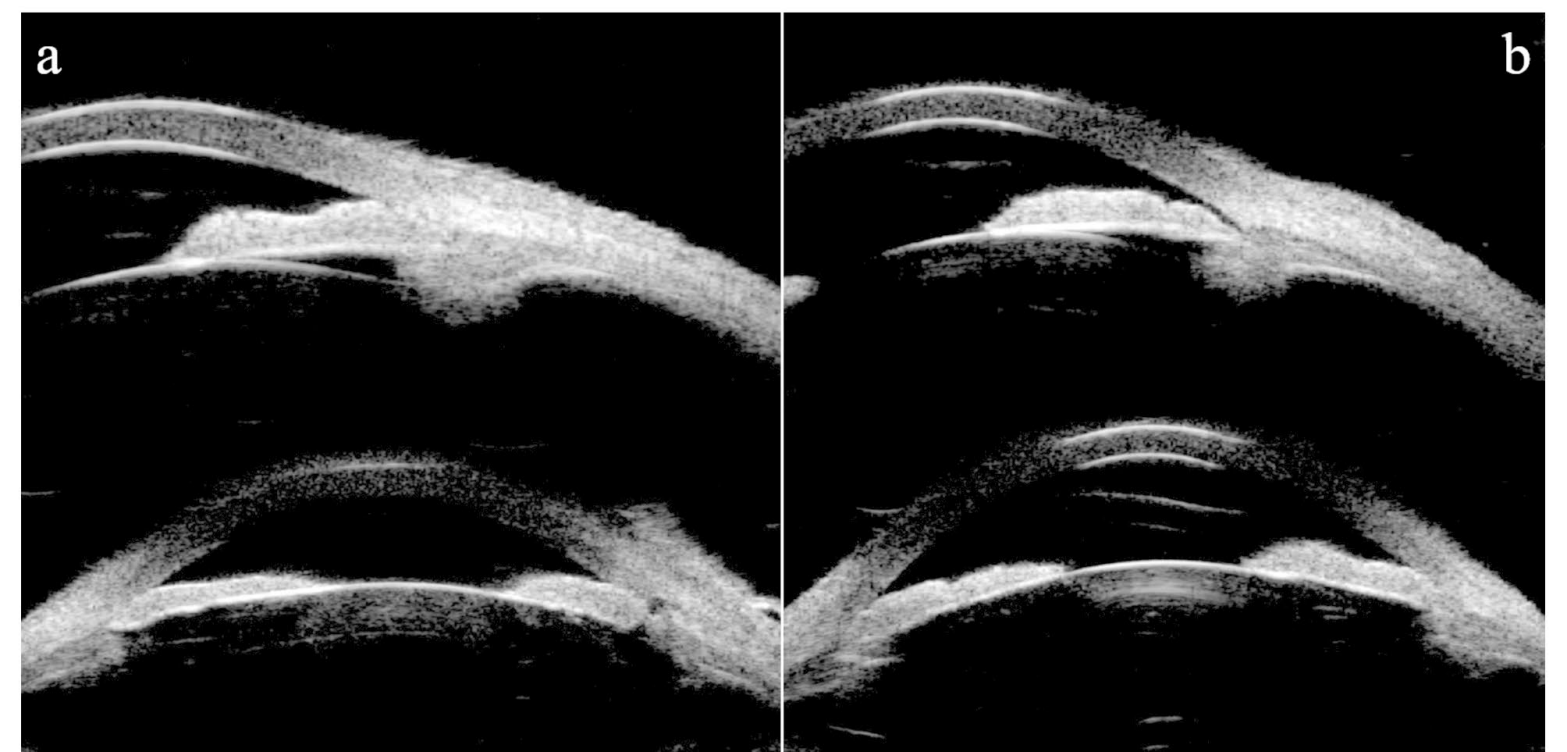
Ultrasound biomicroscopy (UBM) of the bilateral eyes. a: UBM in the right eye showed shallow anterior chamber depth (ACD) (1.64 mm), full circumferential closure of the anterior chamber angle and a peripheral iridotomy; b: UBM in the left eye showed shallow ACD (1.86 mm), close and narrowed anterior chamber angle

Early fluorescein fundus angiography (FFA) of the right eye revealed transmitted hyperfluorescence consistent with retinal pigment epithelium (RPE) atrophy with more extensive areas of choriocapillaris atrophy (Fig. 1c) and late FFA showed weak fluorescence and delay in venous filling in the atrophic areas (Fig. 1e). Macular spectral domain optical coherence tomography showed outer retinal thinning and preretinal hemorrhage at the macula in the right eye (Fig. 2a). Electroretinogram and Electro-oculography (RETI-Port/Scan 21, ROLAND CONSULT Stasche & Finger GmbH, Germany) suggested generalized decrease in photopic and scotopic amplitude in B-wave and A-wave in the right eye. Retinal nerve fiber layer thickness of nasal quadrant was thinner than normal limits in the right eye showed by optical coherence tomography (Spectralis HRA + OCT, Heidelberg Engineering GmbH, Heidelberg, Germany). These examinations of the left eye were within normal limits (Figs. 1d and f and 2b). Ultrasound biomicroscopy (UBM) (Aviso, Quantel Medical, Inc., Bozeman, MT) showed shallow anterior chamber depth (ACD) (1.64 mm), complete circumferential closure of the anterior chamber angle and a peripheral iridotomy in the right eye (Fig. 3a). In addition, UBM in the left eye showed shallow ACD (1.86 mm), close and narrow anterior chamber angle (Fig. 3b).

Laboratory studies including complete blood count, serum electrolytes, serum protein electrophoresis, erythrocyte sedimentation rate, and C-reactive protein were within normal limits. Antinuclear antibody test was negative. Toxoplasma, rubella virus, cytomegalo virus, Hepatitis B surface antigen, human immunodeficiency virus antigen/antibody, T. pallidum antibody and Hepatitis C virus tests were all negative. Chest X-ray ruled out tuberculosis and other active inflammatory process.

The patient had received no specific treatment for RPE or choriocapillaris atrophy and was followed up with IOP at a local clinic. Her IOP were within normal limits during 1-year follow-up period.

## Discussion and conclusions

PPRCA is a rare disease with chorioretinal atrophy in which there is bilateral paravenous RPE atrophy and osteocyte-like pigment clumping [2]. It was originally described by Brown in 1937 in a patient with alopecia [3]. It has been reported to be symmetrical in both eyes, non-progressive or slow progressive and prevalent in males [1, 4]. PPRCA has been reported with cystoid macular oedema [5], intermediate uveitis [6], amblyopia [7] congenital glaucoma and strabismus [8] etc. There is no specific treatment for RPE or choriocapillaris atrophy. In this report, we present a case of unilateral asymmetric PPRCA with AACG. Dilated fundus examination revealed areas of retinochoroidal atrophy and pigment clumping along the retinal veins in the right eye. FFA showed transmitted hyperfluorescence consistent with RPE degeneration with extensive areas of choriocapillaris atrophy. Compared with a recent case of PPRCA with peripheral retinal vascular abnormalities like peripheral capillary non-perfusion, microaneurysms and vascular anastomosis downstream of the pigment clumping in FFA image, [9] RPE degeneration of our patient was more diffuse without obvious peripheral capillary non-perfusion and microaneurysms in peripheral retina. Past medical history of acute attack, shallow ACD and narrow angle showed by UBM, and glaucomatous neuropathy identified by OCT helped to reach the diagnosis of AACG.

The etiology of PPRCA remains unclear, but genetic, developmental and inflammatory causes have been postulated. PPRCA has been previously reported in association with tuberculosis, congenital syphilis, Behçet disease, measles, rubella [1] and Vogt-Koyanagi-Harada disease [10], but its relationship with concomitant diseases has not been reported. Our patient had no evidence of active or previous intraocular inflammation and denied any systematic infectious diseases. Several reported cases have exhibited a familial inheritance. Compound heterozygous mutations of the CRB1 gene were implicated in the etiology of PPRCA [11] and also of retinitis pigmentosa (RP) [12]. Recently reported cases describing patients with unilateral RP and PPRCA in the fellow eye further supported the possibility of shared genetic etiologies [13]. Further genetic testing is needed for our patient and more case studies are required to investigate the role of gene mutation in the occurrence of PPRCA.

Previous researchers suggested that PPRCA was typically symmetrical in bilateral eyes and rarely affects the macula and central vision. A recent study in a large cohort, however, found that 40% of cases exhibited asymmetry in fundus findings [14]. Unilateral PPRCA, as seen in our patient, is exceedingly rare. Cheung reported a patient who had unilateral PPRCA with no history of trauma or a previous inflammatory disease [15]. Bozkurt et al. described a patient of PPRCA and unilateral focal atrophic lesions in his mother and sister [16]. Guillermo et al. described a case of unilateral PPRCA secondary to chronic inflammation with presumed tuberculous uveitis [6]. To the best of our knowledge, this is the first case of unilateral PPRCA with AACG which had no signs of existing or past inflammation. In addition, in our case, the preretinal hemorrhage in the macular region may be caused by the decompression syndrome. Sudden lowering of IOP induces an abrupt increase in retinal intravascular flow which may overwhelm the capillary resistance, resulting in rupture of the retinal capillaries. This phenomenon is commonly referred to as ocular decompression retinopathy [17].

AACG was known to occur in eyes with specific anatomy, such as short axial length, anterior segment crowding and narrow anterior chamber angle. The reason for concurrent manifestation of the PPRCA and AACG remains unclear. Sun et al. described a 55-year-old female who had bilateral PPRCA with AACG and posterior subcapsular cataract, which was the only reported case presented with PPRCA and AACG. Comparison between the two patients are summarized in Table 1. They hypothesized that PPRCA triggered RPE and choroidal atrophy, making eyes less tolerant to high IOP than average glaucomatous eyes and speculated that PPRCA might be an incomplete manifestation of RP, and the mechanisms of co-occurrence of AACG was similar to that of RP complicated by the later disease [18]. Xu et al. found patients with primary angle-closure glaucoma (PACG) associated with RP had the same biometric parameter characteristic as the patients with chronic PACG and acute PACG, suggesting that RP may have a coincidental relationship with angle-closure glaucoma [19]. Recent studies have demonstrated that the association between RP and PACG could be explained by nanophthalmos, cataract, lens subluxation and zonular insufficiency in RP, which are all predisposing factors of PACG [20, 21]. Our patient had no obvious signs of cataract or other symptoms and the pathogenesis of PPRCA with AACG is not yet clearly understood.

**Table 1:** Demographics, Previous History, and Ophthalmic Examination of Patients with PPCRA and AACG

	Patient in this case	Patient in Sun et al.
Age (years)	50	55
Gender	Female	Female
Clinical Symptoms	Vision loss and elevated IOP	Distention and vision loss in right eye
Past medical history	No remarkable systemic, ocular disease and trauma history and not taking any medication	No history of night blindness, ocular or systemic diseases or any other infammatory and infection diseases
Eye	Right eye	Both eyes
BCVA	20/500	20/100 in bilateral eyes
IOP (mmHg)	16	18 in OD and 37 in OS
Anterior Segment	A diffuse superior subconjunctival filtering bleb, a peripheral iridotomy at 9 o’clock, clear cornea, a deep anterior chamber and a clear lens	Shallow anterior chamber and posterior subcapsular cataract
Fundus	Grayish retinochoroidal atrophy and osteocyte-like pigment clumping lesions along the retinal veins and peripapillary preretinal hemorrhage	Retinal choroidal atrophy distributed along the large retinal veins, with large choroidal vessels visible around the optic disc

IOP, intraocular pressure; BCVA, best corrected visual acuity; OD (oculus dexter), the right eye; OS (oculus sinister), the left eye

In summary, we present a rare case of unilateral PPRCA accompanied with AACG. To the best of our knowledge, this is the first such case to be reported.

## Data Availability

The data analyzed during the current study are not publicly available but are available from the corresponding author on reasonable request.

## Abbreviations

AACG: acute angle-closure glaucoma
ACD: anterior chamber depth
EOG: electrooculography
ERG: electroretinogram
FFA: fluorescein fundus angiography
PPRCA: pigmented paravenous retinochoroidal atrophy
IOP: intraocular pressure
OCT: optical coherence tomography
PACG: primary angle-closure glaucoma
RP: retinitis pigmentosa
RPE: retinal pigment epithelium
UBM: ultrasound biomicroscopy
